# Microtubule organization changes severely after mannitol and n-butanol treatments inducing microspore embryogenesis in bread wheat

**DOI:** 10.1186/s12870-021-03345-3

**Published:** 2021-12-09

**Authors:** E. Dubas, A. M. Castillo, I. Żur, M. Krzewska, M. P. Vallés

**Affiliations:** 1 The Franciszek Górski Institute of Plant Physiology, Polish Academy of Sciences, Niezapominajek 21, 30-239 Kraków, Poland; 2 Estación Experimental de Aula Dei, Consejo Superior de Investigaciones Científicas (EEAD-CSIC), Avda Montañana 1005, 50059 Zaragoza, Spain

## Abstract

**Background:**

A mannitol stress treatment and a subsequent application of n-butanol, known as a microtubule-disrupting agent, enhance microspore embryogenesis (ME) induction and plant regeneration in bread wheat. To characterize changes in cortical (CMT) and endoplasmic (EMT) microtubules organization and dynamics, associated with ME induction treatments, immunocytochemistry studies complemented by confocal laser scanning microscopy (CLSM) were accomplished. This technique has allowed us to perform advanced 3- and 4D studies of MT architecture. The degree of MT fragmentation was examined by the relative fluorescence intensity quantification.

**Results:**

In uni-nucleated mannitol-treated microspores, severe CMT and EMT fragmentation occurs, although a complex network of short EMT bundles protected the nucleus. Additional treatment with n-butanol resulted in further depolymerization of both CMT and EMT, simultaneously with the formation of MT aggregates in the perinuclear region. Some aggregates resembled a preprophase band. In addition, a portion of the microspores progressed to the first mitotic division during the treatments. Bi-nucleate pollen-like structures showed a high MT depolymerization after mannitol treatment and numerous EMT bundles around the vegetative and generative nuclei after n-butanol. Interestingly, bi-nucleate symmetric structures showed prominent stabilization of EMT.

**Conclusions:**

Fragmentation and stabilization of microtubules induced by mannitol- and n-butanol lead to new configurations essential for the induction of microspore embryogenesis in bread wheat. These results provide robust insight into MT dynamics during EM induction and open avenues to address newly targeted treatments to induce ME in recalcitrant species.

**Supplementary Information:**

The online version contains supplementary material available at 10.1186/s12870-021-03345-3.

## Background

Doubled haploid (DH) plants constitute a valuable tool in plant breeding as well as in cytogenetics, genetics, and basic research. In recent years there has been a resurgence of research on DH plant production and many efforts have been made to develop efficient protocols (for a review, see [1–3]). Among the various techniques available, microspore embryogenesis (ME) is one of the most efficient (for a review, see [4, 5]). In ME, the trigger is usually a stress treatment that causes the microspores to deviate from the normal gametophytic pathway towards a sporophytic development under *in vitro* culture conditions. Microspores differentiate and form embryo-like structures (ELS), which finally regenerate haploid or double into DH plants. Different stress treatments have proven to be efficient for the induction of ME, including temperature shock, carbohydrates and nitrogen starvation, high pH, etc. In addition, the combination of diverse stresses and/or other compounds, such as microtubule-disrupting agents, membrane permeabilizers, or epigenetic modifiers have also been effective [6–10].

The microtubule (MT) cytoskeleton network plays a fundamental role in response to stress, as its dynamic nature allows its reorganisation following environmental and developmental stimuli [11, 12]. Changes in the cytoskeleton contribute to regulate cell division, cell polarity, the cell wall formation, intracellular transport, and autophagy (for recent reviews, see [13–15]). In ME stress induction, the enlargement of the microspore, the nucleus migration to the centre in ‘star-like’ morphology (SLS), and a preprophase band (PPB) formation preceding the symmetric division depends upon the organization and dynamics of both cortical microtubules (CMT) and endoplasmic microtubules (EMT) [16–19].

In bread wheat (*Triticum aestivum* L.), stress treatments with low temperature or mannitol have been described as the most efficient treatments to trigger ME [20, 21]. Mannitol is a non-metabolizable sugar that added to the medium lowers its water potential, thus it is considered as an osmotic stress inducer, although with a specific downstream response (for a review, see [22]). In ME induced by mannitol a multidimensional response to stress is activated, which involves an essential reorganization of metabolic pathways and activation of cytoprotective mechanisms [23–25]. Accompanying these changes, it has been proposed that mannitol could also act as an anti-microtubule agent in barley microspore embryogenesis induction [26]. Although changes in cytoskeleton dynamics in mannitol acclimation have been proved in other systems [27], no evidence has been provided that the effect of mannitol on ME induction is mediated by changes in MT.

The involvement of the cytoskeleton in ME induction has generated great interest in evaluating the trigger effect of the application of tubulin-targeting agents, e.g., colchicine, cytochalasin D or n-butanol [6, 17, 28]. In particular, the application of the primary alcohol n-butanol (n-butyl alcohol) after a mannitol stress treatment has been shown to greatly enhance ELS production and green plant regeneration in anther cultures of bread wheat and in low-responding cultivars of barley [28–30]. ME productiveness was also increased when n-butanol was applied after cold treatment in anther culture of maize and isolated microspore culture of barley [30, 31] but not as efficiently as in wheat. To our knowledge, no studies on the efficiency of n-butanol on isolated microspores cultures of bread wheat are available. In dicots, ME was also induced by n-butanol in *Borago officinalis* L. [32].

The effect of n-butanol on plant MT has been studied in diverse systems, mainly associated with its capacity to activate phospholipase D (PLD) and decrease the production of phosphatidic acid (PA) [33]. In this sense, an extensive modification of the cytoskeleton was described in BY-2 tobacco cells and Arabidopsis seedlings after n-butanol treatment, with changes in structure and/or dynamics of CMT and EMT [34, 35]. To our knowledge, only one analysis has been focused on the effect of n-butanol on MT dynamics during ME induction [36]. In that study, n-butanol alone or in combination with cold stress treatment in maize had a uniform effect showing complete and reversible depolymerisation of CMT, while the EMT surrounding the nucleus remained relatively intact. However, it is known that different embryogenic pathways could coexist after induction of ME in different species [37, 38], thus a more complex effect of n-butanol might be expected.

To accurately determine the effect of mannitol and n-butanol treatments on MT organization and dynamics during EM induction in bread wheat, a ‘whole mount’ immunolocalization and confocal laser scanning microscopy (CLSM) analysis was performed. 3D and 4D image reconstructions allowed to obtain more reliable information. A quantitative study of the fluorescence intensity of labelled MT was also accomplished. Here, we demonstrate that mannitol-induction produces depolymerisation of both CMT and EMT, variably and correlated with the type of structure present in the suspension. The additional application of n-butanol is characterised by the induction of MT bundles. A model of MT distribution in uni-nucleate microspores in response to both treatments has been postulated. To our knowledge, this is the first report showing discrete effects of mannitol and n-butanol on CMT and EMT in wheat ME induction.

## Results

**Fig. 1:**
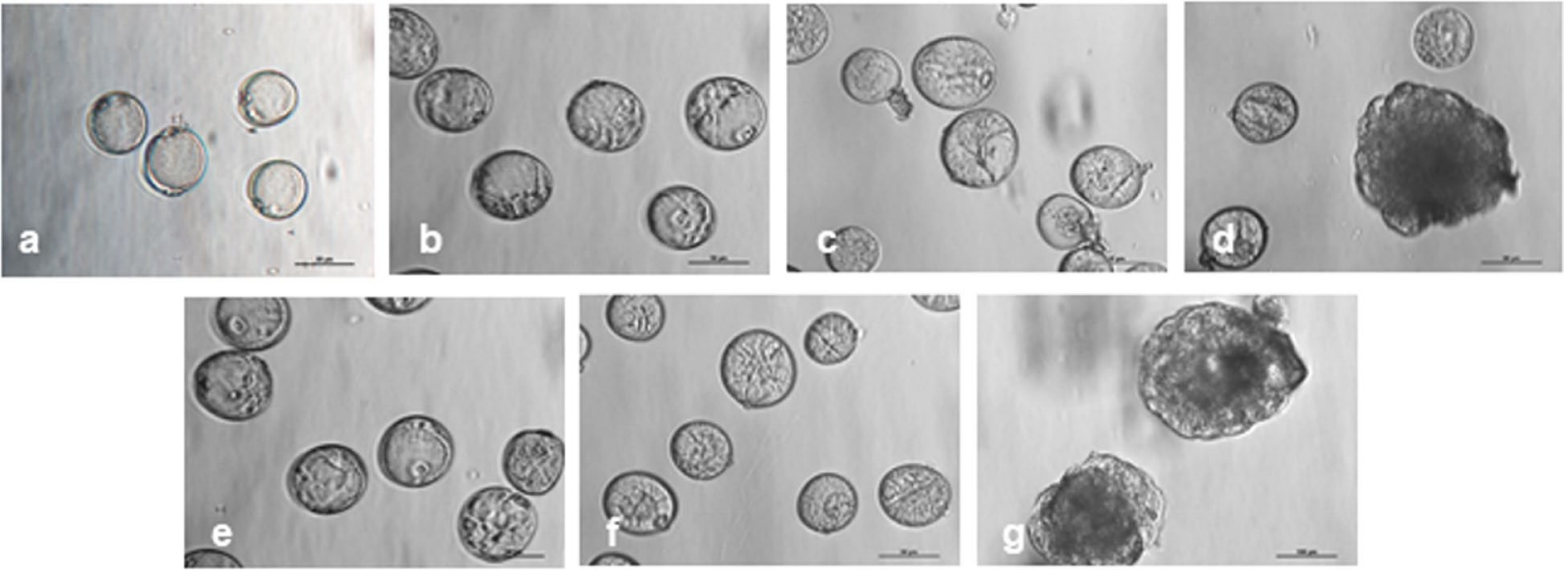
Wheat isolated microspore cultures on OVPCM medium. **a** Isolated microspores from fresh anthers (FM). **b-d** Microspores isolated after a 0.7 M mannitol (MAN) treatment (**b**) and after 3 and 15 days of culture (**c** and **d**, respectively). **e-g** Microspores isolated after mannitol and n-butanol (MANB) treatment (**e**) and after 3 and 15 days of culture (**f** and **g**, respectively). Globular pro-embryos observed after 15 days of culture from anthers treated with MAN (**d**) or with MANB (**g**)

Cultures of bread wheat isolated microspores used in this study were characterized morphologically and by their DH plant production efficiency to verify the effect of a mannitol or a mannitol plus n-butanol treatment. Microspores isolated from fresh anthers (FM) were predominantly at the mid-late uni-nucleate stage, with a large central vacuole and the nucleus located near the sporoderm between the operculum and its opposite site (Fig. 1a). After treatment with 0.7 M mannitol for five days at 25°C (MAN), most microspores showed a vacuole partially fragmented by cytoplasmic strands and the nucleus located on the opposite side of the operculum (Fig. 1b). In microspores treated with mannitol and additionally with 0.2% n-butanol for 4 h (MANB), similar morphologies were observed, although some microspores showed the nucleus in a central position and the fragmented vacuole in the typical star-like morphology (SLS; Fig. 1e). After three days of culture in OVPCM, significant numbers of SLS microspores were observed in both treatments (Figs. 1c, f). Embryogenic structures released from the exine wall were visible after 10-12 days of culture in MAN (Fig. 1d), and between 8 and 10 days in MANB. At 15 days of culture the n-butanol treatment produced a 2-fold higher number of pro-embryos than the mannitol treatment (Figs. 1d, g). Differences between MAN and MANB were also observed in the efficiency of microspores cultures (Table 1). Up to 1.9 and 2.3-fold increase in the number of embryos (N Emb) and green plants (N Green Pl) was observed with MANB.

**Table 1:** Microspore embryogenesis efficiency in the bread wheat cv. Pavon

Treatment	N Emb	N Green PI	N albino PI	Green PI [%]	Reg [%]
MAN	76.5 b*	46.0 b	17.1 a	78.2 b	82.1 a
MANB	143.6 a	105.5 a	16.6 a	86.4 a	79.2 a

Mannitol (MAN); Mannitol and n-butanol (MANB); The number of embryos/10^3^ microspores (N Emb); The number of green plants/10^3^ microspores (N Green Pl); The number of albino plants/10^3^ microspores (N Albino Pl); The percentage of green plants/total plants (Green Pl); The percentage of plant regeneration/embryo (Reg). Values followed by the same letter within each variable are significantly different (*P*≤0.05)

**Scheme 1:**
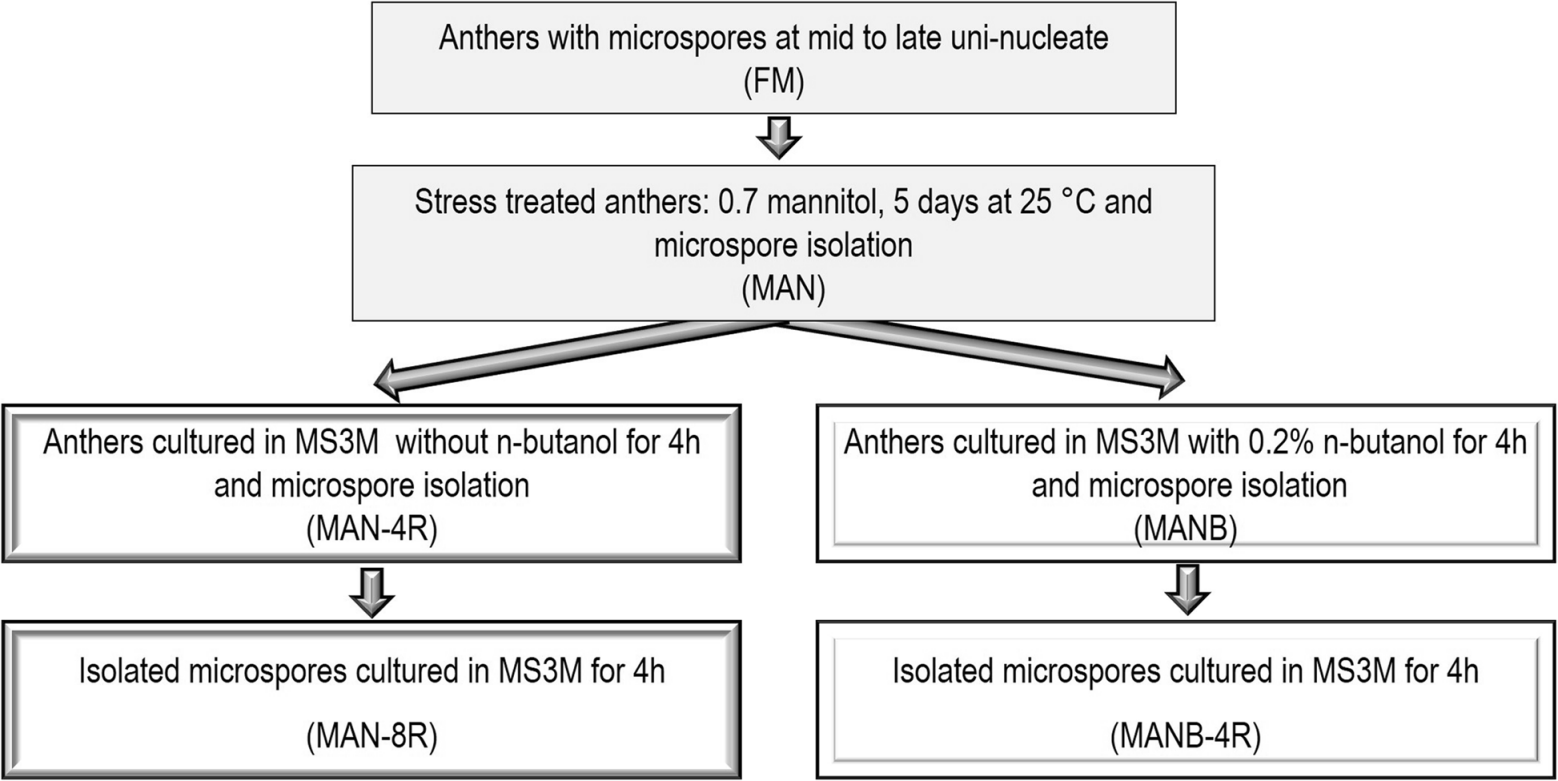
Schematic design of the experiment

The effect of ME inductive treatments on the MT cytoskeleton was examined in microspores isolated immediately after MAN and MANB treatments and compared to fresh microspores (FM), and microspores after 4 and 8 hours of recovery from treatments (MAN-4R, MAN-M8R and MANB-4R) in MS3M medium (Scheme 1). To characterize the structure and organization of MT a whole mount immunolocalization with anti α-tubulin antibodies and confocal laser scanning microscopy (CLSM) was performed. The study was completed with a quantitative study of the fluorescence intensity of labelled MTs and 3D and 4D CLSM image reconstructions of all optical sections. CMT and EMT were studied separately in each stage of differentiation.

### Mannitol and mannitol plus n-butanol treatments change the relative fluorescence intensity of MT in microspores induced to embryogenesis

**Fig. 2:**
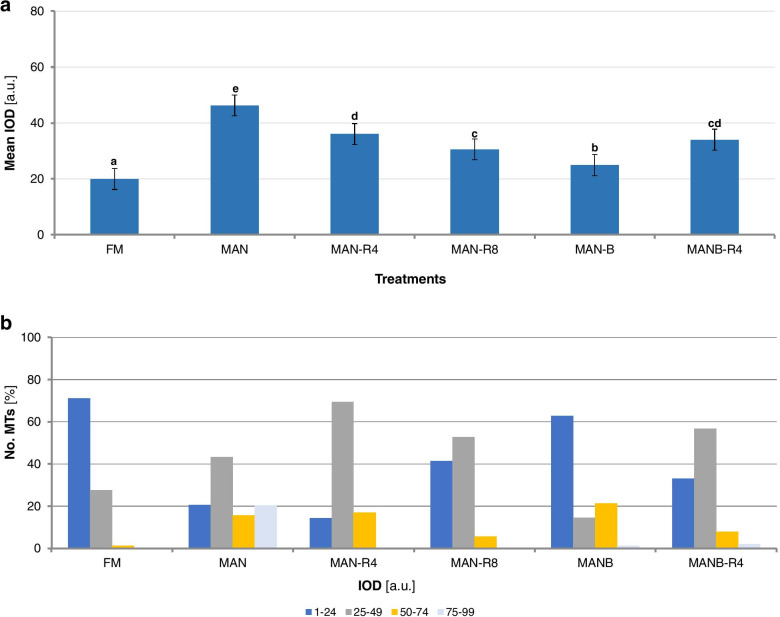
The mean of MT fluorescence intensity and categories allow expressing the degree of MT polymerization in microspores. **A** The mean IOD at 488 nm excitation [a.u.] of MT in fresh microspores (control; FM), exposed to the 0.7 M mannitol stress treatment (MAN) or mannitol and n-butanol treatment (MANB), and during MAN or MANB stress recovering in MS3M medium at 4 or 8 hours (MAN-R4, MAN-R8 and MANB-R4, respectively). Analysis of variance was carried out using GLM procedure of SAS. The Duncan method (α≤0.05) was used for mean separation. Treatments followed by the same letter are not significantly different (*P*≤0.05). **B** Distribution of IOD categories [a.u.] as a function of the polymerization of MT in FM microspores, after MAN, MAN-R4, MAN-R8, MANB, and MANB-R4

Fluorescent imaging and quantitative analysis were implemented to measure changes in α-tubulin fluorescence in uni-nucleated microspores (Fig. 2). Significant differences in mean fluorescence intensity, expressed as integrated optical density (IOD), were observed among samples (Fig. 2a). Fresh microspores (FM) showed the lowest IOD (19.9 a.u.) whereas those treated with mannitol (MAN) the highest (51.7 a.u.). Interestingly, the structures after 4 - 8 hours of recovering from mannitol (MAN-R4 and MAN-R8) had lower values than MAN (31.9 a.u. and 30.8 a.u., respectively). MANB caused almost a 2-fold decrease in IOD (24.9 a.u.) as compared with MAN. Reported values for MANB-R4 (34.0 a.u.) were similar to those obtained for MAN-R4 and MAN-R8.

The measurements of the relative fluorescence intensity were grouped into categories and reflected a steady increase in IOD for all tested samples (Fig. 2b). The last category was made for the most intensively labelled MT appeared as thick bundles. In FM, most of the MT belonged to class 1 (71.0%, Fig. 2b), representing MT as thin filaments. MAN treatment induced thicker MT bundles since class 1 represented, on average, only 20.2%. The rest of MT with higher fluorescence intensity was classified as follows: class 2 (42.1%), class 3 (15.2%) and class 4 (19.9%). IOD of MT in structures collected after 4 hours recovery from MAN (MAN-R4), with class 2 and 3 representing 69.2% and 16.93%, respectively, indicated the disassembly of MT bundles. The same trend was observed after the next 4 hours of recovering (MAN-R8), as class 1 and class 2 represented 94.3% of MT (Fig. 2b). The fluorescence intensity of microspores additionally treated with n-butanol (MANB) indicated the MT disassembly as the fluorescence was mainly concentrated in class 1 (62.8%), however thick bundles were also present with a 21,3% in class 3 (Fig. 2b). Finally, after 4 hours of recovering from n-butanol (MANB-R4), 89.9% of MT were found in classes 1 and 2, with class 2 as the most representative (56.2%). Curiously, this pattern was similar to that observed after MAN treatment recovering phase (MAN-R8).

### Mannitol treatment causes MT disorganization and fragmentation

The ‘whole mount’ α-tubulin immunolocalization technique connected with the special software analysis allowed creating a direct 4D volume reconstruction from the 3D CLSM images. Having such a high-quality image analysis tools, the assessment of MT configuration after the MAN and MANB and during recovery phases have been done.

**Fig. 3:**
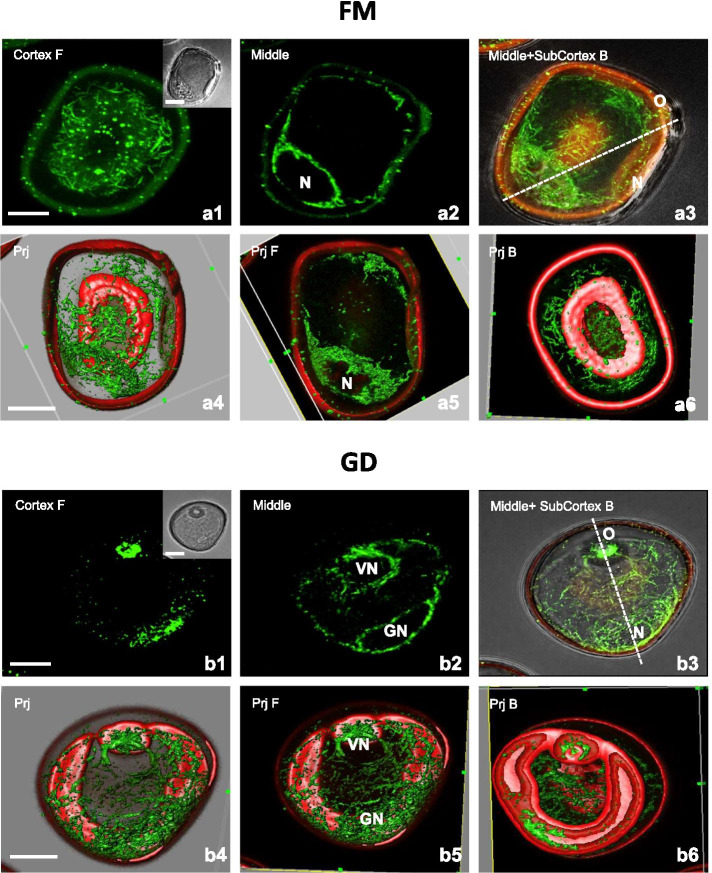
MT configuration in microspores at the early stages of gametophytic development in bread wheat. **A1-A6** Fresh microspore (FM) at uni-nucleate stage. **A1** Randomly oriented long thin bundles of CMT adjacent to the microspore wall. **A2** A dense network of EMT resembling a nest-like structure around located nucleus (N) and in the thin layer of cytoplasm. **A3** A nest-like structure around the N formed by endoplasmic and subcortical MT. **A4-A6**. 3D model of the subcellular volume of the same structure. **A4** Superior visualisation axis model presenting a very dense network of CMT and EMT. **A5** MT tightly associated with the nucleus (N). Volumetric ortho-slicer view from the forward (**A5**) and opposite site of the microspore model (**A6**). **B1-B6** Gametophytic development (GD): Successive optical sections from the bi-cellular pollen grain with the small generative cell close to the sporoderm wall and a large vegetative cell. **B1** CMT close to the sporoderm. **B2** An array of EMT demarcating the generative cell. The generative nucleus (GN) and the vegetative nucleus (VN). **B3** Endoplasmic and subcortical MT parallel and randomly arranged to the long axis of the GN as well as MT within the generative cell under the plasmalemma bordering the vegetative cell. (**B4-B6**) 3D model of the subcellular volume of the same structure. **B4** Superior visualisation axis model, presenting a very dense network of CMT and EMT. **B5** MT tightly associated with the VN and the GN. EMT of the vegetative cell and the generative cell radiated from the perinuclear regions and formed reticulate networks resembled two different basket-like structures for GN and VN, respectively. Volumetric ortho-slicer view from the forward (**B5**) and opposite site of the pollen model (**B6**). Cortex F - CMT; z-series projected as a maximum intensity projection from cortex optical sections collected from the front side (F) of microspore. Middle - optical section at the middle of the z-series. Middle+SubCortex B – Endoplasmic and subcortical MT; z-series projected as a maximum intensity projection from middle and subcortical optical sections collected from the back side (B) of microspore. Inserts on the upper-right corner in the first panels show a corresponding image in the differential interference contrast (DIC). The dashed line indicates the axis between the operculum (O) and the opposite pole. Prj – z-series projected as a maximum intensity from the front side (Prj F) or from the back side (Prj B) of microspore. Green fluorescence (Alexa 488) shows α-tubulin. Inserts on the right in the first panel show a corresponding transmission image. Red fluorescence of nuclei is caused by PI staining. Red autofluorescence of exine. Scales =20 μm

Freshly isolated microspores (FM) showed a complex network of long and thin bundles of the CMT randomly oriented (Figs. 3a1, a4, a6). Similarly, a dense network of thin EMT resembling a nest-like structure was detected around the nucleus in the narrow layer of cytoplasm (Fig. 3a2-a5). Next, after the first pollen mitosis, bicellular pollen grains included a small generative cell (GC) located close to the sporoderm, and a vegetative cell (VC) that occupied most of the pollen volume. At that stage, most cortical and subcortical MT of the vegetative cell were found near the sporoderm (Fig. 3b1, b3-b6). Moreover, EMT of the VC and the GC radiated from the perinuclear regions and formed reticulate networks that resembled two different basket-like structures (Fig. 3b2-b5). Moreover, parallel and random arrangement of subcortical and endoplasmic MT to the long axis of the generative nucleus (GN) as well as MT within the GC under the plasmalemma bordering the VC determined the GC lens-shaped (Fig. 3b2-b6).

**Fig. 4:**
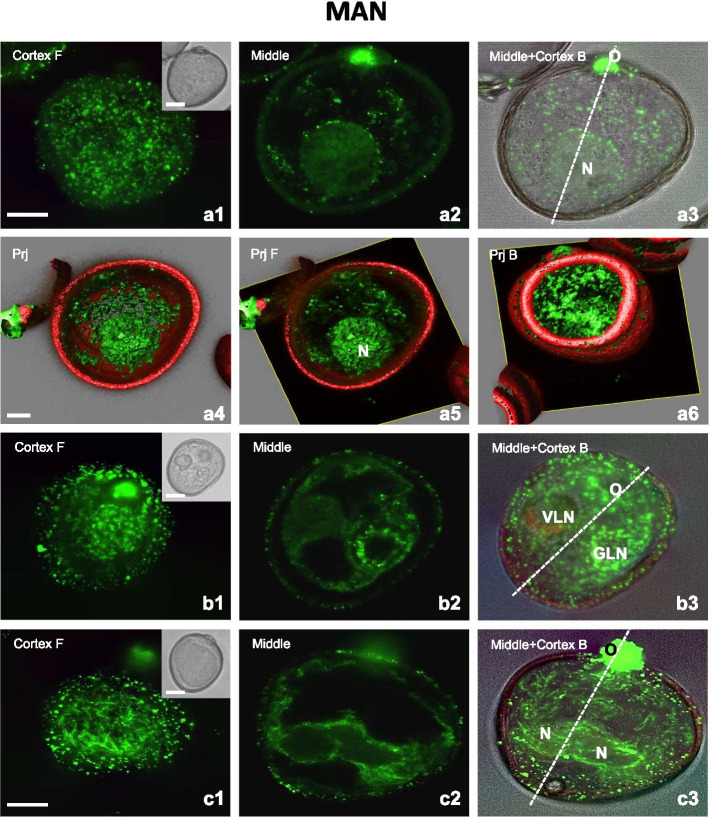
Mannitol induces changes in MT orientation and length in embryogenic microspores. **A1-A6** Late uni-nucleate microspore. Fragmented and disorganized MT in the (**A1, A3**) cortical and subcortical cytoplasm. **A2, A3** Multiple and short EMT bundles around the nucleus (N). **A4-A6** 3D model of the subcellular volume of the same structure. Only scarce and very short CMT and a very dense network of EMT around the nucleus are visible. **A4** Superior visualisation axis model presenting MT tightly associated with the nucleus (N). Volumetric ortho-slicer view from the forward (**A5**) and opposite site of the microspore model (**A6**). **B1-B3** Bi-nucleate pollen-like structure resembling the product of asymmetric gametophytic mitosis with the vegetative-like nucleus (VLN) and generative-like nucleus (GLN). **B1** Scarce and dot-like CMT. **B2-B3** A slightly denser network of scant EMT around the GLN. **C1-C3** Bi-nucleate structure with similar nuclei (N). **C1** Single and mainly short CMT oriented with a preference. **C1-C3** Some long and longitudinal cortical CMT, subcortical MT and EMT around both nuclei forming a basket-like structure. Short and wavy EMT located away from the nuclei and the opposite pole to the operculum (O). Cortex F - CMT; z-series projected as a maximum intensity projection from cortex optical sections collected from the front side (F) of microspore. Middle - optical section at the middle of the z-series. Middle+Cortex B – Endoplasmic and cortical MT; z-series projected as a maximum intensity projection from middle and cortical optical sections collected from the back side (B) of microspore. Inserts on the upper-right corner in the first panels show a corresponding image in the differential interference contrast (DIC). The dashed line indicates the axis between the operculum (O) and the opposite pole. Prj – z-series projected as a maximum intensity from the front side (Prj F) or from the back side (Prj B) of microspore. Green fluorescence (Alexa 488) shows α-tubulin. Red fluorescence of nuclei is caused by PI staining. Red autofluorescence of exine. Scales =20 μm

After MAN treatment, both the late uni-nucleate microspores and bi-nucleate structures have been identified. These bi-nucleate structures either were pollen-like (resembling the product of asymmetric gametophytic division) or embryogenic consisting of two similar nuclei. Prominent changes in the CMT and the EMT in all these types of structures were observed (Fig. 4) in comparison with control (Fig. 3).

In the late uni-nucleate microspores and bi-nucleate pollen-like structures (with the generative-like nucleus GLN and the vegetative-like nucleus VLN), considerable CMT fragmentation and disorganization occurred to the extent that dotted structures appeared (Fig. 4a1, a3, b1, b3). Multiple and short EMT were visualised in the perinuclear region of uni-nucleate microspore constituting a dense network that surrounded the entire nucleus, located at the opposite site to the operculum (Figs. 4a2-a3, a4-a6). Some of these EMT radiated from the nuclear envelope (Fig. 4a2, a5). More loosely packed MT fragments were visible across the microspore cytoplasm (Fig. 4a2-a3, a5). In the bi-nucleate pollen-like structures, a network of scant EMT fragments only around the GLN was observed, whereas individual EMT radiated from the VLN to the cytoplasm (Fig. 4b2-b3). Finally, in structures with similar nuclei, short CMT were oriented with a preference, and longer longitudinally oriented CMT were observed in the vicinity of the nuclei (Fig. 4c1, c3). Located in an almost central position, sister nuclei were surrounded by long EMT forming a framework of thick bundles that appeared to be connected to the plasma membrane (Fig. 4c2). Moreover, short, and wavy EMT were equally visible in the endoplasm (Fig. 4c3).

### Recovery from mannitol treatment favours the rebuilding and disassembling of MT

**Fig. 5:**
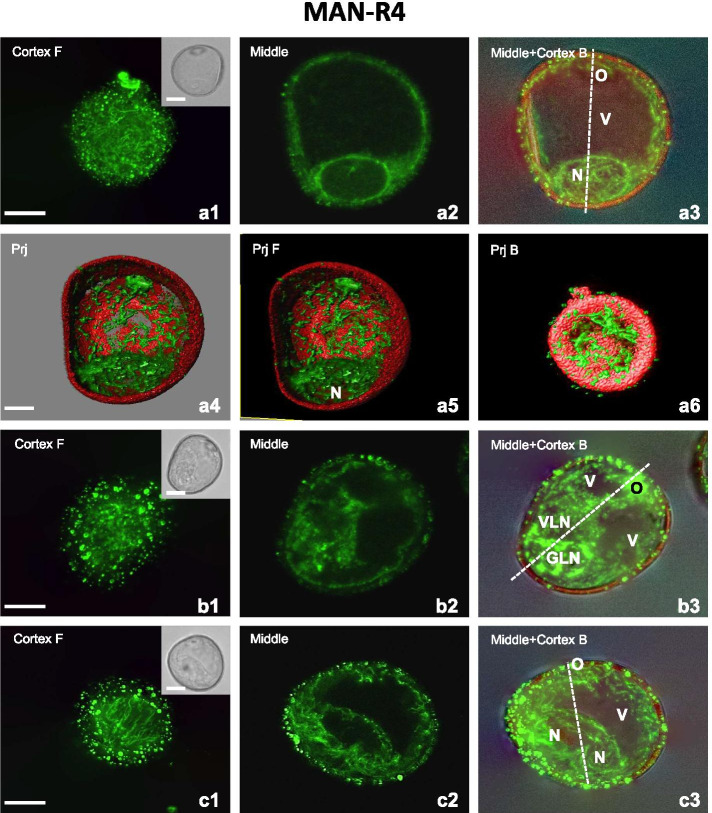
Mannitol stress recovery (MAN-R4) relates to MT regeneration in uni-nucleated microspores and bi-nucleate structures. **A1-A6** Late uni-nucleate microspore. Microspore with a large vacuole (V) and nucleus (N) located close to the exine. **A1** Randomly orientated thin and long CMT. **A2** Long EMT arranged in thin bundles forming a net-like structure wrapped around the N. **A3** Long, single, and thin EMT in the cytoplasmic layer underlying the exine. **A4-A6** 3D model of the subcellular volume of a similar structure. MT recovery observed in the endoplasm, where long and thin EMT bundles assembling a more complex structure causing association of the nucleus with the sporoderm. Superior visualisation axis model (**A4**). Volumetric ortho-slicer view from the forward (**A5**) and opposite site of the microspore model (**A6**). **B1-B3** Bi-nucleate pollen-like structure with the generative-like nucleus (GLN) and vegetative-like nucleus (VLN) and small vacuoles (V) fragmented by cytoplasmic strands. **B1** Short fragmented CMT. **B2-B3** Short EMT in the cytoplasm and in the proximity of the VLN and thick bundles of EMT surrounding the GLN. **C1-C3** Bi-nucleate embryogenic structure with similar nuclei (N) and a large vacuole (V). **C1** Both, disoriented and longitudinally oriented CMT. **C2** Subcortical MT seem to irradiate towards nuclei. **C2-C3** Both, short and long EMT thin bundles forming a basket-like structure surrounding both nuclei. Cortex F – CMT; z-series projected as a maximum intensity projection from cortex optical sections collected from the front side (F) of microspore. Middle – optical section at the middle of the z-series. Middle+Cortex B – Endoplasmic and cortical MT; z-series projected as a maximum intensity projection from middle and cortical optical sections collected from the back side (B) of microspore. Inserts on the upper-right corner in the first panels show a corresponding image in the differential interference contrast (DIC). The dashed line indicates the axis between the operculum (O) and the opposite pole. Prj – z-series projected as a maximum intensity from the front side (Prj F) or from the back side (Prj B) of microspore. Green fluorescence (Alexa 488) shows α-tubulin. Red fluorescence of nuclei is caused by PI staining. Red autofluorescence of exine. Scales =20 μm

The effect of mannitol on MT was clearly reversible since the recovery and repositioning of MT occurred in treated microspores 4 hours after being transferred to the MS3M culture medium (MAN-R4; Fig. 5). In uni-nucleate microspores, CMT, and subcortical MT were thin and long with random orientation (Fig. 5a1, a3, a6) whereas, in two-nuclei pollen-like structures, CMT were still short and dot-like (Fig. 5b1). In microspore-derived structures with two similar nuclei, thin and long CMT were oblique to the main axis and subcortical MT seem to irradiate towards nuclei (Fig. 5c1-c3).

MT recovery was also observed in the endoplasm. In uni-nucleate microspores, the EMT wrapped around the nucleus and formed a net-like structure (Figs. 5a2-a3, a4-a5). Moreover, some EMT were arranged in long and thin bundles assembling a more complex structure, causing association of the nucleus with the sporoderm (Fig. 5a2-a3, a4-a5). On the contrary, there was a low level of recovery in bi-nucleated pollen-like structures (Fig. 5b2-b3). Thus, only depolymerised EMT were observed in the cytoplasm and the proximity of the VLN (Fig. 5b3) while a limited number of thick bundles surrounded the GLN (Fig. 5b3). In the bi-nucleated structures with similar nuclei, organization of EMT was comparable to those observed after mannitol treatment. The EMT mainly assembled as thick and thin, short bundles. Moreover, in the same structure, thin but long EMT surrounded the two sister nuclei to form a framework resembled the basket-shaped structure (Fig. 5c2-c3). And despite this similarity in the assembly, more MT, encircling nuclei and connecting them to the plasma membrane have been also observed (Fig. 5c2-c3).

**Fig. 6:**
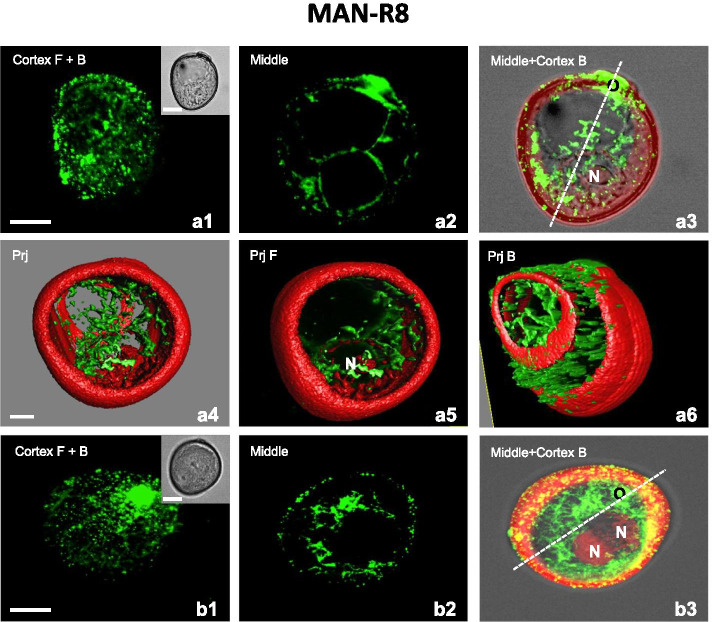
Mannitol stress recovery (MAN-R8) relates to continuous MT regeneration and reorganization in embryogenic microspores. **A1-A6** Uni-nucleate star-like structure (SLS). **A1** Fine CMT, excluding in the vicinity of the nucleus where radial CMT assembled into thicker bundles. **A2** Long strands of EMT in the cytoplasm, transecting the vacuole to the plasma membrane. **A3** EMT strung together to form long and thick strands in the vicinity of the nucleus (N). **A4-A6** 3D model of the subcellular volume of a similar structure. Superior visualisation axis model (**A4**). Long strands of EMT encircled the nucleus (N). Volumetric ortho-slicer view from the forward (**A5**) and opposite site of the microspore model (**A6**). **B1-B3** Bi-nucleate embryogenic structure. **B1** Fine and short CMT without preferential orientation. **B2** EMT assembled into long and thick bundles in the vicinity of the sister nuclei. **B3** The radial EMT network extends out to the cell cortex. Cortex – CMT; z-series projected as a maximum intensity projection from cortex optical sections collected from the front (F) or from the back (B) side of microspore. Middle – optical section at the middle of the z-series. Middle+Cortex B – Endoplasmic and cortical MTs; z-series projected as a maximum intensity projection from middle and cortical optical sections collected from the back side (B) of microspore. Inserts on the upper-right corner in the first panels show a corresponding image in the differential interference contrast (DIC). Prj – z-series projected as a maximum intensity from the front side (Prj F) or from the back side (Prj B) of microspore. Green fluorescence (Alexa 488) shows α-tubulin. Red fluorescence of nuclei is caused by PI staining. Red autofluorescence of exine. Scales =20 μm

The effect of MT reorganization was even stronger after 8 hours of culture (MAN-R8), which resulted in the formation of many SLS structures and structures with two similar nuclei (Fig. 6). In the SLS, the CMT were observed as fine, longitudinally arranged filaments, excluding in a small region close to the nucleus where MT constituted a radial assembly (Fig. 6a1, a6). EMT strung together to form long strands that encircled the nucleus (Fig. 6a2-a3, a4-a5). In the bi-nucleate structures with similar nuclei, the CMT were also observed as fine, longitudinally arranged filaments (Fig. 6b1). The EMT established a dense radial network of long and thick bundles that extended out to the cell cortex from the surface of the sister nuclei (Fig. 6b2-b3).

### An additional n-butanol treatment induces MT fragmentation

**Fig. 7:**
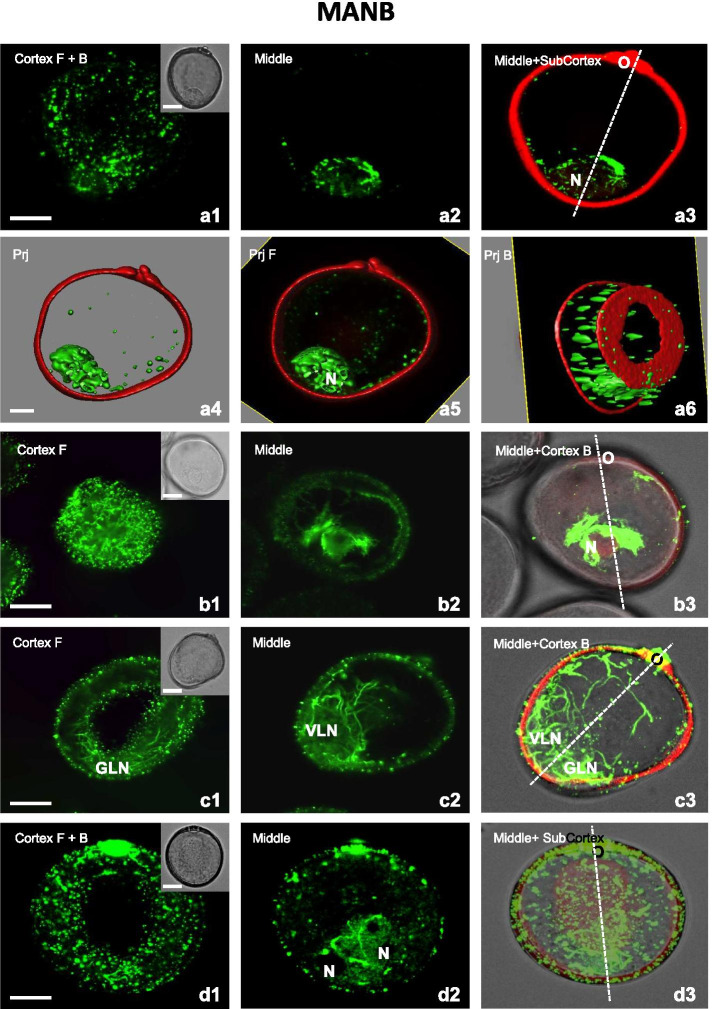
A mannitol and n-butanol treatment (MANB) affects CMT and EMT in microspores and bi-nucleate structures. **A1-A6** Late uni-nucleate microspore. **A1** Few CMT fragments assembled mainly into very short thin bundles. **A2-A3** Long, thick, and wavy aggregates of EMT in the cytoplasm in the vicinity of the nucleus (N). **A4-A6** 3D model of the subcellular volume of a similar structure. Few CMT fragments. Long, thick, and wavy aggregates of EMT observed in the cytoplasm around the nucleus (N). Superior visualisation axis model (**A4**). Volumetric ortho-slicer view from the forward (**A5**) and opposite site of the microspore model (**A6**). **B1-B3** Late uni-nucleate microspore. **B1** Numerous, short and non-preferentially oriented CMT assembled mainly into thick bundles. **B2-B3** Thick bundles of MT (EMT and subcortical) encircling the midplane of the nucleus, resembling a PPB-like structure at the initial phase of formation. **C1-C3** Bi-nucleate pollen-like structure with the generative-like nucleus (GLN) and the vegetative-like nucleus (VLN). **C1** Few and highly fragmented CMT. **C2-C3** Long, thick, and wavy aggregates of EMT forming a network with the VLN and the GLN. **D1-D3** Bi-nucleate embryogenic structure. **D1** Fine and very short CMT bundles without preferential orientation. **D2-D3** Thick bundles of EMT encircling the midplanes of the sister nuclei (N). Cortex - CMT; z-series projected as a maximum intensity projection from cortex optical sections collected from the front (F) or from the back (B) side of microspore. Middle - optical section at the middle of the z-series. Middle+(Sub)Cortex B - Endoplasmic and (sub)cortical MT; z-series projected as a maximum intensity projection from middle and cortical optical sections collected from the back side (B) of microspore. Inserts on the upper-right corner in the first panels show a corresponding image in the differential interference contrast (DIC). The dashed line indicates the axis between the operculum (O) and the opposite pole. Prj – z-series projected as a maximum intensity from the front side (Prj F) or from the back side (Prj B) of microspore. Green fluorescence (Alexa 488) shows α-tubulin. Red fluorescence of nuclei is caused by PI staining. Red autofluorescence of exine. Scales =20 μm

In all types of structures present in suspension, both CMT and EMT were affected by additional treatment with 0.2% n-butanol for 4 hours (MANB) (Fig. 7). In the late uni-nucleate microspores, bundled or non-bundled MT arrays were detected. Some few, short, unoriented CMT were observed (Fig. 7a1), together with longitudinally oriented CMT assembled mainly in thin bundles (Fig. 7a6). The spaces between bundles were filled with fine and very short non-bundled CMT (Fig. 7b1). Similarly, a highly fragmented CMT were observed in bi-nucleated structures (Figs. 7c1, d1).

Long, thick and wavy aggregates of fragmented EMT were observed in the cytoplasm around the nucleus in the late uninucleate microspores (Figs. 7a2-a6). In some microspores these thick bundles together with subcortical MT encircled the midplane of the nucleus, resembling the very early phase of a PPB-like formation (Figs. 7b2-b3). In bi-nucleate pollen-like structures, long, thick, and wavy aggregates of EMT were also observed around the two nuclei (VLN, GLN), although the network of EMT around the GLN showed a higher density (Figs. 7c2-c3) and no PPB-like structures were visible (Fig. 7c3). In contrast, in structures with two similar nuclei, thick, and long bundles of MT were observed close to both nuclei (Fig. 7d2-d3).

### Recovery from n-butanol treatment promotes MT reconstruction and thickening around the nucleus

**Fig. 8:**
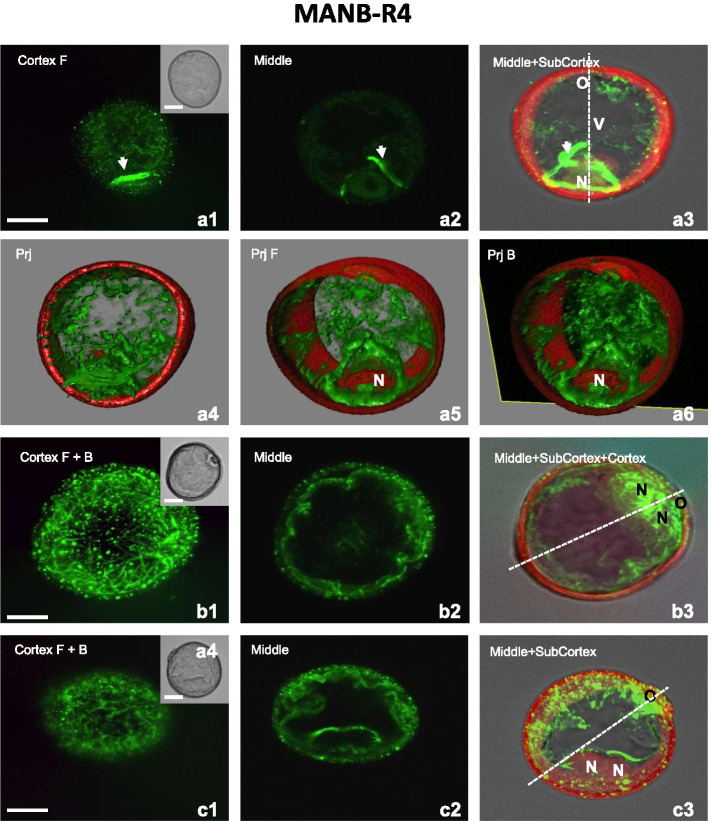
Mannitol and n-butanol stress recovery (MANB-R4) relates to continuous MT regeneration in embryogenic microspores. **A1-A6** Late uni-nucleate microspore with a large vacuole (V). **A1** Most cortical and subcortical MT assembly into long, thin bundles without a preferential orientation. **A2-A3** Some thick bundles of CMT encircling the midplane of the nucleus (N) resembling a PPB-like structure. Scarce, fine, and very short EMT in the remaining cytoplasm above the nucleus (**A3**). **A4-A6** 3D model of the subcellular volume of a similar structure. Some thick bundles of CMT formed a ‘ring’ around the nucleus (N) of microspore resembling a narrow PPB-like structure. Superior visualisation axis model (**A4**). Volumetric ortho-slicer view from the forward (a5) and opposite site of the microspore model (**A6**). **B1-B3, C1-C3** Bi-nucleate embryogenic structures. **B1** Numerous long and thin CMT. **B2-B3** Reticulate network of long EMT extending to the cortex around the sister nuclei (N). **C1** Thin and very short CMT. **C2-C3** Dense network of MT in the cytoplasm at the opposite pole to the nuclei (N). Long bundles of EMT encircling both nuclei and growing in the direction of the cortex. Cortex - CMT; z-series projected as a maximum intensity projection from cortex optical sections collected from the front (F) or from the back (B) side of microspore. Middle - optical section at the middle of the z-series. Middle+(Sub)Cortex B - Endoplasmic and (sub)cortical MT; z-series projected as a maximum intensity projection from middle and cortical optical sections collected from the back side (B) of microspore. Inserts on the upper-right corner in the first panels show a corresponding image in the differential interference contrast (DIC). The dashed line indicates the axis between the operculum (O) and the opposite pole. Prj - z-series projected as a maximum intensity from the front side (Prj F) or from the back side (Prj B) of microspore. Green fluorescence (Alexa 488) shows α-tubulin. Red fluorescence of nuclei is caused by PI staining. Red autofluorescence of exine. Scales =20 μm

A characterization of MT was performed 4 h after the transfer of n-butanol treated microspores to the culture medium (MANB-4R, Fig. 8). Cortical and subcortical MT bundles in all structures were thin and long, mostly without a preferential orientation (Fig. 8a1, b1, c1). However, some thick bundles of CMT formed a ‘ring’ around the nucleus of the microspore (Fig. 8a1-a2, a4-a6) or radiated from similar nuclei (Fig. 8b1-b2).

In microspores, scarce and fine EMT were detected at the same time (Fig. 8a2-a3, Additional file S1). Contrary, in structures with two similar nuclei, long bundles of EMT formed a denser reticulate network anchoring both nuclei to the plasma membrane (Fig. 8b2-b3), but thick bundles could also be observed close to the nuclei (Fig. 8c2-c3). Cytoplasmic EMT grew in the direction of the plasma membrane and sometimes formed visible sites of EMT-cortex attachment (Fig. 8b2-b3, c2-c3).

## Discussion

Microspore embryogenesis induction is generally triggered by a stress treatment that generates a reorganization of microspore structure, and finally leads to a sporophytic development. In bread wheat, microspores can be efficiently triggered to undergo ME by a mannitol treatment [10, 20], but the number of embryogenic structures increases with an additional treatment with n-butanol (a MT-depolymerizing agent) [28, 29]. Several morphological features that characterize the switch to the sporophytic pathway are associated with the MT cytoskeleton dynamics (for a review, see [39]). However, it is unknown how the reorganization of MT occurs as a function of the type of stress applied in wheat ME induction. In this work, high-resolution temporal and spatial data of the MT state by CLSM analysis of α-tubulin coupled with powerful 4D multidimensional visualization, has allowed us to define the MT dynamics associated with ME-inducing treatments and to propose a model that could support the design of new ME induction strategies.

The mannitol ME-inducing treatment (MAN) is a combination of sugar starvation and osmotic stress. Mannitol has been commonly used in *in vitro* assays to mimic drought and particularly to study the acclimation response (for a review, see [22]). In these studies, mainly at hypocotyl epidermal or root cells, changes in MT dynamics contributed to maintain cell shape and survival under increased osmotic pressure [27, 40]. The application of mannitol to induce ME involves a complex stress response with a broad range of molecular mechanisms and regulatory networks involved [24]. This study shows that mannitol treatment also had an intense and diverse effect on microspores MT dynamics.

**Scheme 2:**
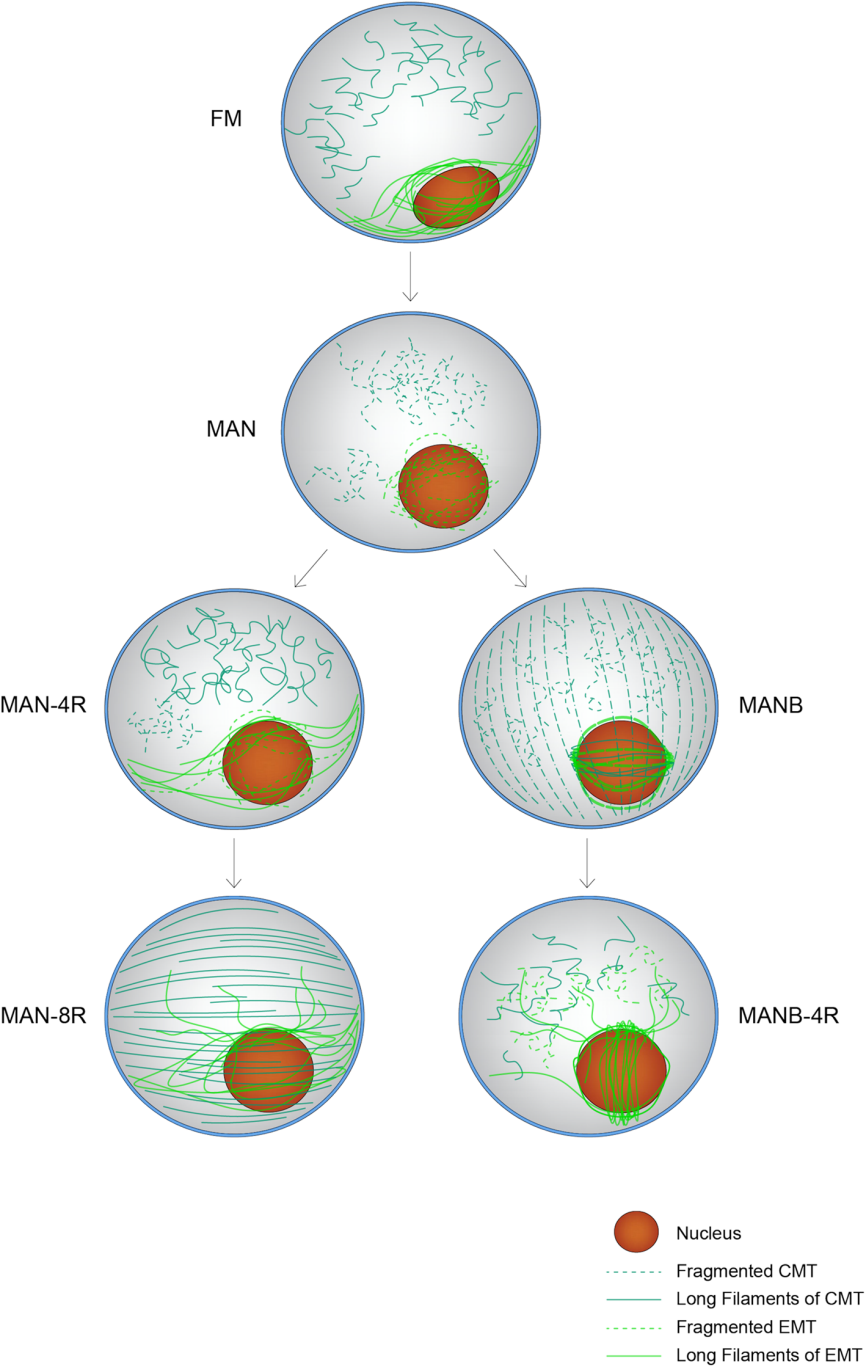
The two-dimensional model of MT configuration in the sphere-shaped uni-nucleate microspores induced to embryogenesis. Most representative unique directions of visible MT are represented: cortical MT near the cell surface (CMT, dark green) and endoplasmic MT (EMT, light green) in the cytoplasm including the vicinity of the nucleus. Freshly isolated microspores (FM) were subjected to stress treatments with 0.7 M mannitol for five days at 25°C (MAN) or with mannitol and additionally with 0.2% n-butanol for 4 h (MANB) to induce embryogenesis and were subsequently cultured in MS3M medium for 4 or 8 hours for recovering (MAN-4R, MAN-8R, MANB-4R). In FM, long CMT bundles were randomly oriented while long EMT determined a net-like structure wrapped around the nucleus. After treatments, the uni-nucleate microspores showed characteristic pattern of MT orientation and fragmentation. MAN affects the MT length and orientation. While CMT were fragmented and disorganized, short EMT bundles formed a protective nest-like structure around the nucleus. After MAN recovering (MAN-4R), microspores displayed randomly oriented, fine, and longer CMT bundles as compared with MAN and long EMT determined a net-like structure around the nucleus. In MAN-R8, thin and long CMT bundles were oriented, and thickened EMT bundles were found in the vicinity of the nucleus. The additional treatment with n-butanol (MANB) resulted in the fragmentation of CMT and EMT bundles. As a result, thick bundles of subcortical MT and wavy aggregates of EMT in the midplane of the nucleus were observed. This MT configuration resembled a PPB-like structure at the initial phase of formation. Recovering of MANB (MANB-4R) involved a lengthening of both, the CMT and the EMT. A band of subcortical MT encircled the midplane of the nucleus to be like a PPB-like structure. Moreover, few thick EMT bundles surrounded the nucleus

Fresh uninucleate microspores show a well-characterized MT network in a strong polarized cell with a single large vacuole and a lens-shaped nucleus in a proximal position opposite to the operculum (Fig. 3, FM; Scheme 2 – FM) [41]. Single CMT were found in the thin layer of cytoplasm and numerous EMT connected the nucleus to the plasma membrane, helping to maintain its position. MT are particularly important in the next stages of pollen development as an asymmetric cell division is essential for correct GC and VC differentiation (Fig. 3d, g) [42]. Microspore MT network is severely altered after MAN treatment. A general trend towards increased MT assembly was observed, as stated by the increase in relative MT fluorescence intensity (2.5 times with respect to FM), and 42.1% of MT corresponded to a medium-low intensity class (Fig. 2). Visualization of MT allows us to associate assembly differences with the state of CMT, EMT and perinuclear EMT. Mannitol causes a severe fragmentation of CMT and their detachment from the plasma membrane, being especially striking in EMT that anchored the nucleus in its position (Fig. 4a; Scheme 2 – MAN). This allows the nucleus to be in a slightly more centered position and to recover a spherical shape, completely enclosed by a complex network of short bundles of EMT, evenly distributed (Fig. 4a; Scheme 2 - MAN). This situation could favour a subsequent symmetrical division and initiate the sporophytic development (for a review, see [39]).

The observed effect of MAN on MT dynamics in wheat differed substantially from that described in maize ME induced by a 10-day cold treatment [36]. In maize, no differences in the MT network with respect to untreated microspores were observed, whereas actin filaments exhibited variations in the organization. We should consider that in the adaptive response to cold stress or osmotic pressure, successive phases of MT depolymerization/polymerization are necessary (for a review, see [13]). Therefore, the unaltered MT observed in maize may correspond to a stabilization phase in a long cold treatment while altered MT network after mannitol could correspond to a prior phase of destabilization. However, it should also be noted that differences in the effect on MT may be due to the different response to ME-inducing treatments between species or even within the same species [6, 20].

Interestingly many factors involved in ME induction, such as calcium, stress-induced proteins or ABA accumulation [43–45] are components of the stress adaptation response. MT can be downstream targets in this adaptive response to stress, but also upstream regulators of stress signalling [46]. Thus, MT participate in propagation of signals as in calcium influx or reactive oxygen species (ROS) production (for review, see [47]) or downstream of protein kinase [48, 49], bioactive phospholipid metabolism [50] and abscisic acid induced signal transduction [51].

After MAN, most microspores do not progress to the first pollen division, but the treatment does not prevent some microspores to divide (Fig. 4). In those pollen-like bi-nucleate (Fig. 4) an arrangement of MT as the expected in the bi-nucleated pollen was observed (Fig. 3d, g), but with signs of MT fragmentation in both cortex and cytoplasm. In barley, it has been described that only 30% of these structures could follow an embryogenic development pathway [37]. Moreover, MAN also induces symmetric microspore division (Fig. 4). Symmetric division is the default division in pollen development when signalling in the cytoskeleton has been modified [42]. Sister nuclei located in an almost central position were surrounded by long EMT connected to the plasma membrane and short CMT were organized with a preferential orientation (Fig. 4). This MT conformation is possibly associated to enhanced stress adaptation by maintaining and reinforcing microtubule integrity and stability [27, 52]. Symmetric microspore division has been described as the most efficient to produce embryo-like structures in ME [53].

MT rearrangements caused by mannitol in uninucleate microspores were reversible upon culture in MS3M culture medium. After 4 hours of recovery (MAN-R4), a significant decrease in the MT assembly was observed (Fig. 2) with the apparent relative fluorescence intensity being mainly in class 2 (69.3%) (Fig. 2). MT disassembly possibly preceded the establishment of a more stable MT configuration [12]. It should be noted that a less dense EMT net-like structure surrounded a spherical nucleus and the MT anchoring the nucleus to the sporoderm was diffuse (Fig. 5, Scheme 2 - MAN-R4). As mentioned above de-anchoring of the nucleus was considered a requirement to allow movement of the nucleus toward a central position to facilitate the symmetrical division (for a review, see [39]). The bi-nucleate pollen-like structures were unrecovered, confirming a high instability. And those structures with sister nuclei presented longer MT and a higher density of MT surrounding the nuclei and connecting them to the plasma membrane (Fig. 5).

The patterns observed after 4 hours recovery were confirmed after 8 hours (MAN-R8), highlighting the initiation of SLS-type uninucleate structures (Fig. 6, Scheme 2 - MAN-R8). SLS is considered as a morphological marker of microspores induced to ME [54], especially in wheat, barley, triticale, and rye [18, 53, 55, 56], although some SLS structures may not successfully develop embryos [55]. In SLS and structures with two similar nuclei, long, thick EMT bundles radiated and extended from the surface of the nuclei to the cell cortex (Fig. 6), possibly associated with the microspore reactivation [57].

The application of the MT depolymerizing agent n-butanol to mannitol-treated microspores (MANB) has been shown to increase the number of embryogenic structures in wheat anther culture [28]. This work described by the first time that the n-butanol treatment doubled the number of green plants in isolated microspores cultures of bread wheat (Fig. 1). These results are comparable with those in anther culture [28]. It is known that application of n-butanol disorganizes MT networks in different plant systems [34, 35]. However, the effect of n-butanol as MT depolymerizing agent in microspore MT was poorly studied.

As expected, the application of n-butanol to mannitol-treated microspores induced a decrease in the IOD parameter, demonstrating extensive depolymerization of MT (62.8% in class 1) (Fig. 2). However, this effect was only observed in CMT and subcortical MT, as the most striking feature of MANB was the presence of MT aggregates in the perinuclear region (Fig. 7, Scheme 2 -MANB). Previous reports in tobacco BY-2 cells had shown that the effect of n-butanol on MT was not restricted to interphase cortical MT, but also to those in preprophase band and phragmoplast [34]. In addition, a combination of n-butanol with mannitol treatment revealed the formation of atypical tubulin bundles in the root tip of *T. turgidum* [58]. Although these accumulated preferentially near the plasmalemma, some were also localized as rod-like near or within the nucleus.

The effect of MANB on EMT observed in this study contrasts with that described in maize ME with a 6-hour treatment alone or after cold treatment, as n-butanol destroyed the CMT but not EMT around the nucleus [36]. These differences could be due to the different states of MT at the time of n-butanol application, caused by the prior stress applied. It is known that n-butanol increased ME efficiency in barley more efficiently after mannitol than after cold treatment [30]. Or it could be a consequence of distinct cytoskeletal construction between species, as proposed by Földesiné-Fúredi et al. [31].

The higher efficiency of ME in bread wheat after n-butanol treatments could be related with further depolymerization of EMT anchoring the nucleus that facilitates nucleus shift to a central position promoting symmetric division (Fig. 7, Scheme 2 -MANB) (for a review, see [39]). In addition, the strong alteration of MT in the perinuclear region could modify the internal structure of the nucleus altering its fate [59, 60]. The formation of bundles in the perinuclear region could also favor the formation of structures associated with the different mechanisms related to sporophytic development. Accordingly, in some uni-nucleate microspores discontinued thick bundles of EMT in the perinuclear zone were observed (Fig. 7, Scheme 2 -MANB), but in others, these thick bundles together with subcortical MT encircled the nucleus, resembling the early formation of a PPB-like structure. In ME, the presence of a PPB-like in the prophase of the first mitotic division defines symmetric division and determines the progress of the microspore towards an embryogenic pathway (for a review, see [39]). Thus, a PPB-like was observed in embryogenic microspores of *Brassica* [16, 18, 19, 61]. However, as n-butanol disrupts the PPB from the cell cortex [34], the structure of the PPB could be affected and be atypical. It should be noted that nuclei with a PPB-like structure were not always in a central position as described in triticale, where the symmetric division was frequently recognized near the sporoderm [18].

Structures with two symmetrical nuclei showed both short and long EMT bundles surrounded the nuclei. Interestingly, in the pollen-like structures, the EMT bundles encircling both the VLN and the GLN were very characteristic (Fig. 7), contrasting with those observed after a single mannitol treatment (Fig. 4). This configuration of moderately thick and flexible MT bundles in the two nuclei may indicate that n-butanol causes the same EMT reorganization in the two cells regardless of cell fate. These results suggest that n-butanol treatment could stimulate mitosis of both vegetative and generative type nuclei, adopting pollen embryogenesis pathways as described by Daghma et al. [37].

Recovery from n-butanol treatment (MANB4) promoted MT reconstruction (Fig. 2). Few longer and thinner bundles of EMT and CMT were observed without preferential orientation, except numerous longitudinal MT assemblies growing toward or away from the nucleus (Fig. 8) similarly to mannitol recovering (Fig. 6). Simultaneously, a long, thick and flexible bundle observed around the nucleus could be an altered narrow PPB structure (Fig. 8, Scheme 2- MANB-R4). However, it should be noted that the first symmetric division in pollen development occurs in the GN, and CMT configuration of germ cell formed a dense open ring structure around GN similar to that observed [62]. As it has been suggested that the ring would facilitate the detachment of the nucleus from the sporoderm and having a protective role during GN migration [62], the observed structure could represent protection of the nucleus rather than a PPB-like.

It is now widely understood that stress perceived at the cell membrane, activates PLD producing phosphatidic acid (PA) and promoting its association with MAD 65-1 (microtubule-associated protein MAD 65-1), facilitating the stabilization and recovery of MT [63, 64]. As n-butanol inhibits PLD-dependent PA production [65], it was assumed that the application of n-butanol would reduce the level of PA and consequently the level of stabilization of MT closed to the sporoderm. However, this study shows that not only microspore CMT but also EMT are susceptible to n-butanol, and that n-butanol mediated the formation of MT bundles in the perinuclear region in ME. Therefore, it cannot be excluded that another n-butanol mechanism of action not related to PA levels was implicated. In this sense, a model of the direct activation of PLD by n-butanol has been proposed [34, 35].

## Conclusions

The results presented provide robust insight into MT dynamics during ME induction in bread wheat. The ‘whole-mount’ α-tubulin immunolocalization technique produces high-quality images that allow advanced 3- and 4D reconstructions of the MT cytoskeleton in uni-nucleate microspores and bi-nucleate structures, making this work highly valuable. The model presented here explains how mannitol causes MT depolymerization by modifying the anchoring of the nucleus to the wall and stabilization of bundles surrounding the nucleus ensuring its protection. This type of arrangement could sustain a positive impact on the survival of microspores/structures and their embryogenic development. In addition, n-butanol disrupted CMT and EMT, highlighting the formation of thick bundles resembling a preprophase band that defines the orientation of symmetric division. However, we cannot discard that these MT bundles could facilitate the migration of the nucleus to a more centered position before division. Although further investigation on the mechanism implicated in the observed MT modifications are needed, this study allows us to address new and more specific treatments affecting MT dynamics as strategies to induce ME in recalcitrant species.

## Materials and methods

The spring bread wheat cultivar Pavon was used since it is highly responsive to ME. Seeds were provided by Instituto Nacional de Investigación y Tecnología Agraria y Alimentaria (INIA) in Madrid (Spain). Seeds of donor plants were sown in a paper pot with a mixture of peat, vermiculite and sand (1:1:1). Plants were vernalized for 5 weeks in a growth chamber at 6 °C, with an 8/16 h dark/light photoperiod and 100 μE m^-2^ s^-1^ light provided by fluorescent tubes (LumiLux Cool Daylight 30W). Plants were transplanted into 15-cm-Ø pots with the same soil mixture described above and cultivated in a growth chamber at 12-14 °C with a 12 h photoperiod and 500 μE m^-2^ s^-1^ light provided by high-pressure metal halide lamps (Phillips Powerone HPI-T Plus 400 W). After 3 weeks, the temperature was increased to 18-21 °C, and the photoperiod was lengthened to 16 h of light. Relative humidity was maintained at 60-65%. An N:P:K (20:20:20) and micronutrient fertiliser was applied once per week (1 g/pot).

### Anther and microspore cultures

Anthers containing most microspores at the mid- to late uninucleate stage (Fresh microspores; FM, Scheme 1) were excised from the flowers and cultured in 0.7 M mannitol, 40 mM CaCl_2_ and macronutrients from FHG medium [66] with 8 g l^-1^ SeaPlaque agarose for 5 days (MAN) (Scheme 1). After stress treatment, the anthers were cultured on MS3M liquid medium [67] for 4 hours without n-butanol (MAN-R4) or with 0.2% n-butanol (MANB), following the protocol described by Soriano et al. [28]. Microspores were isolated from FM, MAN, MAN-R4, and MANB, following the protocol described by Castillo et al. [68], with some modifications: anthers were macerated with a ceramic rod, and all centrifugation steps were performed at 80 ×g for 4 min at 4 °C. Microspores isolated from anther after mannitol and 4 hours of culture in MS3M medium (MAN-R4) and mannitol and 2% butanol (MANB) treatments were cultured in MS3M liquid medium for 4 hours (MAN-R8, and MANB-R4, respectively) (Scheme 1). Culture density was adjusted at 1.3 × 10^5^ microspores ml^- 1^. The microspore cultures on liquid medium were maintained in the dark on a rotary shaker at 160 rpm.

A half volume of the microspore suspension from MAN-R4 and MANB-R4 was used for further morphological characterization of the androgenic response. Therefore, after centrifugation at 80 ×g for 4 min, microspores were cultured in MS3M medium containing 300 g l^–1^ Ficoll 400, which had been previously conditioned for 5 days with 10 ovaries at 25 °C. The ovaries used for pre-conditioned medium were maintained during culture with anthers or microspores (OVPCM) [67]. For characterization of plant production after MAN and MANB, three more microspore isolations were performed with the same batch of plants. The following variables were recorded: number of embryos/10^3^ microspores (N Emb), green plants/10^3^ microspores (N Green Pl) and albino plants/10^3^ microspores (N Albino Pl), percentage of green plants/total plants (Green Pl (%)), and percentage of plant regeneration/embryo (Reg (%)).

### Immunolocalization of microtubules

Samples from FM, MAN, MAN-R4, MAN-R8, MANB and MANB-R4 (Scheme 1) were used for the ‘whole mount’ immunolabeling of microtubules (MT) according to Dubas et al. [61], with substantial modifications to improve microspore collection, cell preservation and antibody penetration.

Samples were collected with special handmade sieves prepared from ‘blue’ pipette tips (5 mm in diameter) with 30 μm Nylon mesh (Celltricks, Partec). Sieves were placed in Eppendorf tubes in a freshly prepared pre-fixative solution containing 1% paraformaldehyde (PFA, Sigma 76240) and 0.025% glutaraldehyde (GA, Sigma 49626) in microtubule stabilisation buffer (MTSB: 50 mM 1,4-piperazinediethanesulfonic acid (PIPES), Sigma P-1851), 5 mM EGTA (Sigma O-3778), and 5 mM MgSO_4_, with pH adjusted to 7.0 with 5 M KOH. The pre-fixative solution was removed from Eppendorf tubes after 10 min, and the samples were re-immersed in MTSB with 3% PFA and 0.025% GA overnight on ice at 4 °C under vacuum (900 Pa). After fixation, the samples were washed in MTSB/0.025% Triton X-100 (5 times, 10 min). Aldehydes were reduced with a mixture of 0.05 M NH_4_Cl and 0.05 M NaBH_4_ for 5 min, and the samples were washed twice again as described above. Next, the cell wall was partially digested with a mixture of 1% cellulase (Ozonuka R10, SERVA 16419), 0.8% pectinase (Sigma P-2401), 0.02% pectolyase (Sigma P-3026) and 0.3% macerozyme (R10, SERVA 28302) in MTSB for 3 hours at 37 °C. Next, the cells were washed 5 x 10 min each with MTSB/0.025% Triton X-100. To enhance microspore permeability, samples were incubated in MTSB with 10% DMSO and 3% Nonidet P-40 for 50 min at room temperature. After rinsing 3 times, a blocking step was performed with 2% BSA in MTSB at 30 °C for 30 min. Primary monoclonal antibody (anti-α-tubulin clone DM1A raised in mouse, dilution 1.1000 Sigma T-9026) was applied overnight at 4 °C in MTSB with 3% BSA in the dark. Microspores were washed 5 times 10 min in MTSB/0.025% Triton, after which the secondary antibody GaM/IgG/Alexa 488 (A-11001 Molecular Probes, dilution of 1:100) was applied in blocking buffer for 3 hours at 37 °C in the dark. Thereafter, the microspores were washed with 0.025% MTSB/0.02% Triton (5 times 10 min) and MilliQ water (5 times 10 min). Finally, the microspores were stored in 0.02% NaN_3_ in PBS.

### Nucleus fluorescent staining

For DNA staining, the samples were incubated in 0.1% propidium iodide (PI, Sigma P-4170) for 15 min, washed in PBS and embedded on slides in Citifluor-glycerol or in 4′,6-diamidino-2-phenylindole x 2HCl (DAPI, Sigma-Aldrich, D-9564; according to Custers et al. [69]; for 15 min, washed in PBS and embedded on slides in Citifluor-glycerol (Citifluor in glycerol, AF2, Enfield Cloisters). Reagent, i.e., DAPI or PI, was added directly to the slide to an equal volume of fresh sample of suspension. Slides were kept in a humid chamber in darkness for at least 15 min before microscopic analysis.

### Microscopic observations of microspores

Microscopic observations of the cytoskeleton were performed under a C1 confocal laser scanning microscope (CLSM) and fluorescence microscope ECLIPSE-E600 (Nikon). CLSM images were collected by the averaging of 4 full scans. Three-dimensional images and z-projections of the cells were obtained by collecting series of approximately 10–30 optical sections in the Z-axis, with each section 0.5–1 μm thick. Fluorescence image stacks were registered in parallel in the 488-nm (green) and 561-nm (red) channels. The series of differential interference contrast (DIC) images were registered in a subsequent scan. The images were acquired and processed using appropriate software, including Imaging system C1, NIS-Elements (AR 2.10 Laboratory Imaging System, Ltd.), Image-Pro Premier 3D 9.3, Photoshop and Corel Photo-Paint X5. For CLSM imaging, single sections or projections of serial optical sections were used (cortical CMT, endoplasmic EMT, or both merged) to localize tubulin and/or nuclei.

Microspore culture were examined under a Nikon Eclipse E600. Images were recorded by a digital camera (Digital Sight DS 5MC) and processed by the NIS-Elements (AR 2.10 Laboratory Imaging System, Ltd.) programme.

### Measurements of MT fluorescence intensity

The relative fluorescence of MTs was calculated after background subtraction, and data were presented as a mean of fluorescence intensity (integrated optical density; IOD) in arbitrary units [a.u.]. Based on fluorescence intensity, the MT were subdivided into four classes with 25 a.u. intervals. The IOD per cell was calculated using the Image-Pro Premier 3D 9.3 software.

### Statistical analysis

Statistical analysis of variables associated with DH production and fluorescence intensity measures (IOD) was performed using SAS software (SAS Institute Inc., Cary, NC, and Version 9.1). Variables expressed as percentage were transformed with square root (x + 0.5) and analysis of variance was carried out using GLM procedure of SAS. The Duncan method (α ≤ 0.05) was used for mean separation.

## Supplementary Information

**Additional file 1: **(**Figure S1**; video) of the 4D volumetric animation of z-series from MANB-R4 microspore collected by CLSM. A 4D volumetric animation of z-series projected as a maximum intensity from MANB-R4 microspore collected by confocal microscopy. Green: microtubules visualized by antibody against α-tubulin, red: nucleus stained with propidium iodide (PI), and autofluorescence of exine. Some thick bundles of CMT formed a ‘ring’ around the nucleus (N) of the microspore resembling a narrow PPB-like structure.

## Data Availability

The datasets generated and/or analysed during the current study are available from the corresponding authors for reasonable request.

## Abbreviations

CLSM: Confocal laser scanning microscope
CMT: Cortical microtubules
DH: Doubled haploid
DIC: Differential interference contrast
DNA: Deoxyribonucleic acid
3D: Three-dimensional
4D: Four-dimensional
ELS: Embryo-like structures
EMT: Endoplasmic microtubules
GC: Generative cell
GLN: Generative-like nucleus
IOD: Integrated optical density
MAD 65-1: Microtubule-associated protein MAD 65-1
MAN: Mannitol
ME: Microspore embryogenesis
MT: Microtubules
PA: Phosphatidic acid
PLD: Phospholipase D
PPB: Preprophase band
ROS: Reactive oxygen species
VC: Vegetative cell
VLN: Vegetative-like nucleus
